# Estimation of effective imaging dose and excess absolute risk of secondary cancer incidence for four‐dimensional cone‐beam computed tomography acquisition

**DOI:** 10.1002/acm2.12741

**Published:** 2019-10-08

**Authors:** Yuki Yuasa, Takehiro Shiinoki, Ryota Onizuka, Koya Fujimoto

**Affiliations:** ^1^ Department of Radiation Oncology Graduate School of Medicine Yamaguchi University Ube Yamaguchi Japan; ^2^ Department of Radiological Technology Yamaguchi University Hospital Ube Yamaguchi Japan

**Keywords:** effective dose, four‐dimensional cone‐beam computed tomography, Monte Carlo simulation, secondary cancer risk

## Abstract

This study was conducted to estimate the organ equivalent dose and effective imaging dose for four‐dimensional cone‐beam computed tomography (4D‐CBCT) using a Monte Carlo simulation, and to evaluate the excess absolute risk (EAR) of secondary cancer incidence. The EGSnrc/BEAMnrc were used to simulate the on‐board imager (OBI) from the TrueBeam linear accelerator. Specifically, the OBI was modeled based on the percent depth dose and the off‐center ratio was measured using a three‐dimensional (3D) water phantom. For clinical cases, 15 lung and liver cancer patients were simulated using the EGSnrc/DOSXYZnrc. The mean absorbed doses to the lung, stomach, bone marrow, esophagus, liver, thyroid, bone surface, skin, adrenal glands, gallbladder, heart, intestine, kidney, pancreas and spleen, were quantified using a treatment planning system, and the equivalent doses to each organ were calculated. Subsequently, the effective dose was calculated as the weighted sum of the equivalent dose, and the EAR of the secondary cancer incidence was determined for each organ with the use of the biologic effects of ionizing radiation (BEIR) VII model. The effective doses were 3.9 ± 0.5, 15.7 ± 2.0, and 7.3 ± 0.9 mSv, for the lung, and 4.2 ± 0.6, 16.7 ± 2.4, and 7.8 ± 1.1 mSv, for the liver in the respective cases of the 3D‐CBCT (thorax, pelvis) and 4D‐CBCT modes. The lung EARs for males and females were 7.3 and 10.7 cases per million person‐years, whereas the liver EARs were 9.9 and 4.5 cases per million person‐years. The EAR increased with increasing time since radiation exposure. In clinical studies, we should use 4D‐CBCT based on consideration of the effective dose and EAR of secondary cancer incidence.

## 1 INTRODUCTION

Cone‐beam computed tomography (CBCT) is widely used in image‐guided radiotherapy (IGRT) to evaluate patients and their anatomical changes and calculate the dose distribution. The accuracy of the CBCT setup has been extensively reported to be superior to two‐dimensional (2D) X‐ray images, because it facilitates observation of bone and soft tissue.^1^, ^2^ However, CBCT images usually exhibit artifacts in the thorax and upper abdominal region produced by patients’ respiratory motion. For tumors in the thorax and abdomen, respiratory motion results in geometric and dosimetric uncertainties when delivering radiotherapy to the target, which presents varying motion patterns and geometric relations during the treatment course.^3^ To cover geometric variations due to respiration, internal target volume (ITV) methods can be used, but large internal margins are required, thus inducing toxicity to normal tissue. To reduce internal margins, motion management for the thorax and upper abdominal regions is important.^4^ Recently, several devices have been developed to manage respiratory motion, with four‐dimensional CBCT (4D‐CBCT) being particularly useful.^5^, ^6^ Some reports demonstrated the accuracy of tumor localization and image quality of 4D‐CBCT.^7^, ^8^ Likewise, our research group has reported the ability of 4D‐CBCT to acquire highly accurate images from a respiratory motion phantom.^9^ Several studies have reported that 4D‐CBCT can be used to manage respiratory motion in clinical cases.^10^, ^11^, ^12^ They stated that 4D‐CBCT can observe the internal margin and the motion of a tumor during treatment with high accuracy, and that 4D‐CBCT can be used for stereotactic body radiotherapy as an IGRT device. Consequently, the number of 4D‐CBCT acquisitions has been increasing in clinical cases.

According to Task Group 75 of the American Association of Physicists in Medicine, the dose should be adjusted to minimize the risk of deterministic injury to normal tissue and inducing cancer or genetic defects. Although it has been reported that the imaging dose for IGRT is smaller than that for treatment, its impact to normal tissue is not negligible.^13^ The imaging dose of IGRT devices has been reported previouly.^14^, ^15^ In particular, the imaging dose for CBCT acquisition is larger than that for other imaging devices. For instance, Kan et al*.* reported an effective imaging dose for CBCT acquisition in the head, chest, and pelvis of 10.26 ± 0.46, 23.56 ± 0.35, and 22.72 ± 0.29 mSv, respectively, which are larger than those required to obtain 2D images or planning CT.^16^ Other studies have reported the imaging dose for CBCT.^17^, ^18^ However, the case of 4D‐CBCT has not been sufficiently addressed, even though its imaging dose is expected to be larger than that of conventional CBCT acquisition, as the time to obtain projection data is longer. Furthermore, 4D‐CBCT parameters, such as gantry rotation time and frame rate, differ from those of conventional CBCT. As 4D‐CBCT is used extensively in clinical scenarios to manage respiratory motion, information on its imaging dose is important from the perspective of health problems managements, such as skin burns, bone marrow suppression, circulatory disease, cataracts and risks of secondary cancer incidence.

Similarly, the risk of secondary cancer incidence can increase with higher imaging doses. Dzierma et al. reported the imaging doses and risks of secondary cancer incidence for several computed tomography (CT) and some CBCT scan sequences.^19^ Although these authors estimated organ doses using thermo‐luminescent dosimeters (TLD) with the use of phantom, clinical studies could not be evaluated. With the exception of this study, few reports have investigated the risk of secondary cancer incidence during 4D‐CBCT acquisitions.

In this study, we estimated the organ equivalent dose and effective imaging dose during 4D‐CBCT acquisition using a Monte Carlo simulation and evaluated the excess absolute risk (EAR) of secondary cancer incidence using the biologic effects of ionizing radiation (BEIR) VII model.

## 2 MATERIALS AND METHODS

### 2.1 Measurements and Monte Carlo simulation for 3D water phantom

The percent depth dose (PDD) at the isocenter and off‐center ratio (OCR) at the depths of 1, 5, and 10 cm along the *x*‐ and *y*‐axes were measured using a 3D water phantom (Blue phantom; IBA Dosimetry GmbH, Schwarzenbruck, Germany) and a 0.13 cm^3^ ionization chamber (CC13 farmer chamber; IBA Dosimetry GmbH, Schwarzenbruck, Germany). The on‐board imager (OBI) mounted on a TrueBeam linear accelerator (Varian Medical Systems, Inc., Palo Alto, CA, USA) was fixed at 0° to measure the PDD and OCRs. The tube voltage and current were set to 125 kV and 40 mA, respectively. The frame rate and X‐ray pulse duration per frame were set to 7 frames/s and 20 ms, respectively. A beam hardening filter made of titanium and a half‐bowtie filter were used for all the measurements. The half‐value layer of the X‐ray tube was 8.90 mm. In addition, the source‐surface distance was 100 cm, and the field size was 26.5 × 19.8 cm^2^ (x1 = 23.9, x2 = 2.6, y1 = 9.9, and y2 = 9.9 cm) at the isocenter. References x1 and x2 were set to right and left, whereas y1 and y2 were set to inferior and superior, respectively. The ionization camber was driven with low‐speed continuous mode to measure the dose profile.

The OBI source was simulated using the EGSnrc/BEAMnrc codes^20^, ^21^ to generate a phase‐space file with a tube voltage of 125 kV, which was constructed with position, direction, charge and energy data of all the particles for arbitrary plane. The phase‐space file was generated at a distance of 70 cm from the focal spot of the X‐ray tube. The directional bremsstrahlung splitting number was set to 20000, and the number of histories was 7 × 10^9^ in a source number of 13 (parallel rectangular beam incident from side). The data on Koch–Motz and the National Institute of Standards and Technology (NIST) were used for bremsstrahlung angular sampling and cross‐sections.^22^, ^23^ The spin effects, photoelectron angular sampling, and atomic relaxations were observed. Rayleigh scattering was not observed. The XCOM‐NIST data were used for photon cross‐sections, and EXACT and PRESTA‐2 were employed for boundary crossing and electron step algorithm. The Bethe–Heitler was used for pair cross‐sections. The field size was 26.5 × 19.8 cm^2^ at the isocenter. The X‐ray tube, tube exit window, blades, beam hardening filter, and half‐bowtie filter were incorporated using the XTUBE, CONSTAK, JAWS, SLABS, and JAWS component modules, respectively. For the transport parameter of EGSnrc, the electron and photon cut‐off energies (ECUT and PCUT) were set to 512 keV and 10 keV, respectively. The generated phase‐space file was used to simulate the PDD at the isocenter and OCRs along the *x*‐ and *y*‐axes. The simulated PDD and OCRs were calculated using the EGSnrc/DOSXYZnrc codes^21^ and compared to the measurements. The PDD and OCR simulations were performed using a water phantom with dimensions of 60 × 60 × 60 cm^3^. The voxel size was 2.5 × 2.5 × 2.5 mm^3^. The water material provided by the International Commission on Radiation Units and Measurements (ICRU) was used for phantom simulation, and the material density of water was 1.0 g/cm^3^.^24^, ^25^ For the transport parameter of DOSXYZnrc, we set the same ECUT and PCUT as for the generation of the phase‐space file. To obtain statistical uncertainty below 1%, the number of histories was 1 × 10^10^ in a source number of 2 (full phase‐space source file).

### 2.2 Calibration of Monte Carlo simulation

The beam output was calibrated using measurements and simulations in accordance with a previous report.^26^ The calibration factor was calculated by comparing the absolute dose measured in a water‐equivalent phantom with the simulated dose under the same geometry as that used for the measurements of absolute dose.

**Figure 1:**
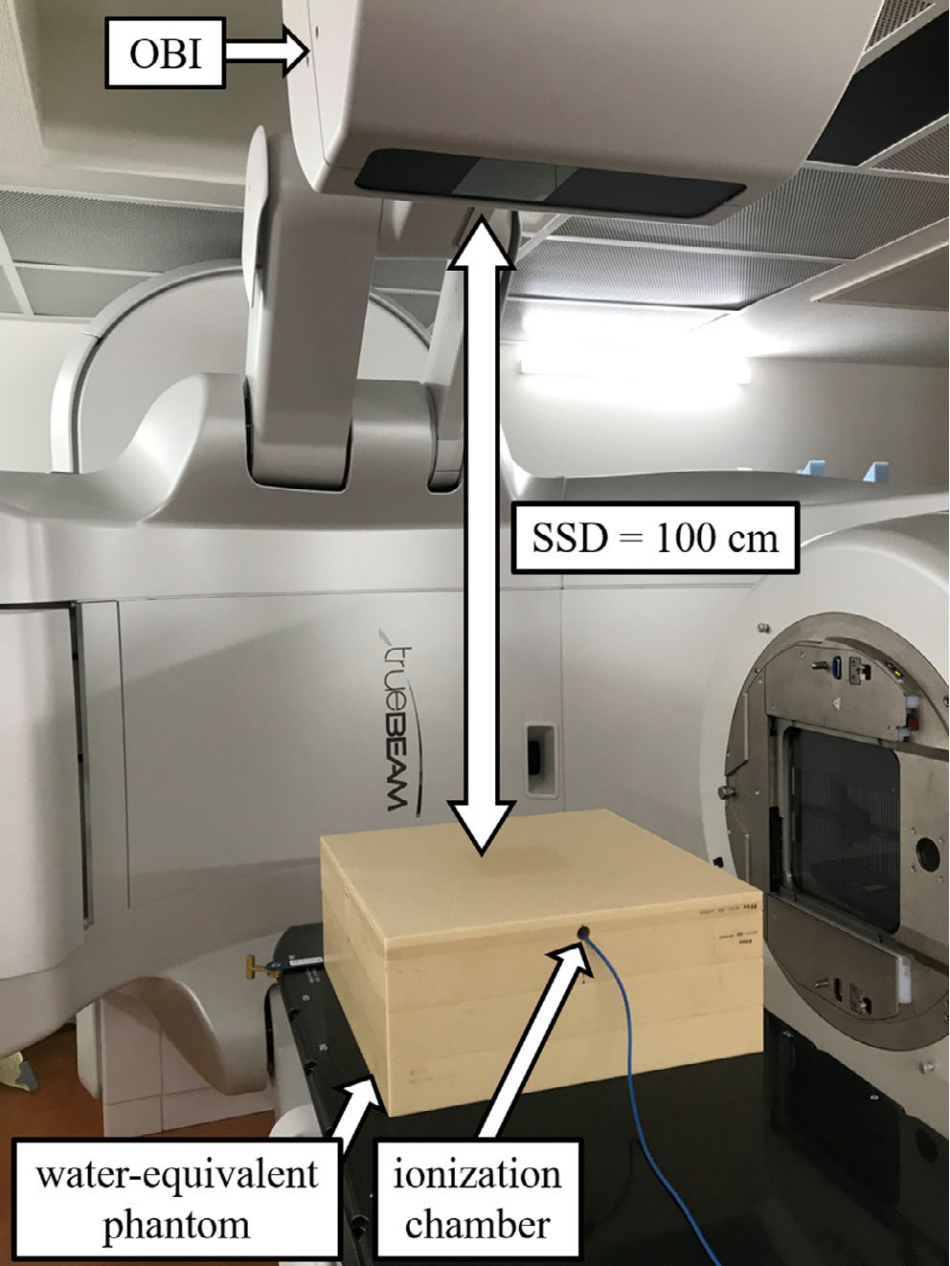
Experimental setup for the measurement of the absolute dose. A phantom with dimensions of 40 × 40 × 17 cm^3^ was positioned at the isocenter with a source‐surface distance (SSD) of 100 cm. The dose to water was measured using a 0.6 cm^3^ ionization chamber. The on‐board imager (OBI) was fixed at 0° to measure the dose.

For the measurements, the absolute dose was measured at a depth of 2 cm for a water‐equivalent phantom with dimensions of 40 × 40 × 17 cm^3^. According to Task Group 61 of the American Association of Physicists in Medicine, the dose to water, *D*_w_, was determined using a 0.6 cm^3^ ionization chamber (PTW30010 Farmer Chamber; PTW Freiburg GmbH, Freiburg, Germany).^27^ The water‐equivalent phantom was positioned at the isocenter with a source‐surface distance (SSD) of 100 cm (Fig. 1). The OBI was fixed at 0° to measure the dose. The tube voltage of the OBI was set to 125 kV with a half‐bowtie and titanium filter. The tube current‐time product (mAs) value was set to 200 mAs. The tube current was 257 mA and the exposure time was 777 ms.

For the Monte Carlo simulation, the simulated dose, *D*_MC_, was calculated based on the same geometry as that used for the measurement of absolute dose. The ECUT and PCUT were set to the same values as those used for the simulations described in section 2.A. The calibration factor, $f_{\text{cal}}$, was defined as.

(1) $$f_{\text{cal}}=\frac{D_{w}}{D_{\text{MC}}\times A_{\text{cal}}\times T_{\text{cal}}}$$

where *D*_w_ is the dose measured using the ionization chamber, *D*_MC_ is the dose calculated using Monte Carlo simulation, $A_{\text{cal}}$ is the tube current of OBI and $T_{\text{cal}}$ is the exposure time of OBI. We set $A_{\text{cal}}$ to 257 mA and $T_{\text{cal}}$ to 777 ms.

### 2.3 Patient simulations

The characteristics of lung and liver cancer patients are summarized in Tables 1 and 2, respectively. Fifteen patients who underwent radiotherapy at our institution were used to simulate the dose distributions using the DOSXYZnrc codes^21^ from a generated phase‐space file. We determined the number of patients using a sample size formula with a 95% confidence interval and a margin of 5%. This study was approved by the institutional review board.

**Table 1:** Lung cancer patients’ characteristics.

Patient No.	Sex	Height (cm)	Weight (kg)	BMI (kg/m^2^)	Tumor location
1	F	147.3	49.2	22.7	RUL
2	M	157.0	45.6	18.5	RML
3	F	156.2	51.1	20.9	LLL
4	M	164.0	49.0	18.2	RML
5	F	138.6	31.4	16.3	RLL
6	M	158.6	55.3	22.0	RUL
7	F	137.0	40.6	21.6	RLL
8	F	146.0	55.0	25.8	RLL
9	M	N/A	N/A	N/A	LLL
10	M	158.5	63.3	25.2	RUL
11	M	169.3	48.3	16.9	LLL
12	M	166.0	69.6	25.3	RLL
13	F	147.8	51.5	23.6	LUL
14	F	150.7	48.9	21.5	RLL
15	F	166.8	69.0	24.8	LLL

BMI, body mass index; F, female; LLL, left lower lobe; LUL, left upper lobe; M, male; RLL, right lower lobe; RML, right middle lobe; RUL, right upper lobe.

**Table 2:** Liver cancer patients’ characteristics.

Patient No.	Sex	Height (cm)	Weight (kg)	BMI (kg/m^2^)	Tumor location
1	M	156.0	55.7	22.9	S4
2	F	146.7	49.2	22.9	S1
3	F	145.3	46.4	22.0	S8
4	M	159.2	54.0	21.3	S5
5	M	166.2	63.1	22.8	PVTT
6	M	159.5	49.2	19.3	S3
7	M	159.2	50.5	19.9	S4, S7
8	M	168.8	49.5	17.4	S7
9	M	162.7	46.9	17.7	S3, S4
10	M	168.5	59.1	20.8	PVTT
11	M	155.9	64.0	26.3	S8
12	F	146.3	68.6	32.1	S2
13	M	162.4	53.0	20.1	S2
14	M	165.7	86.4	31.5	S7
15	M	162.1	52.8	20.1	PVTT

BMI, body mass index; F, female; M, male; PVTT, portal vein tumor thrombosis; S, liver segment.

For the simulation, the planning CT was performed with 20‐slice CT (SOMATOM Definition AS Open; Siemens Healthcare, Erlangen, Germany) in a full‐scan mode. The resulting images were converted to the material and mass density format (egsphant file) using an in‐house MATLAB (MathWorks, Natick, MA, USA) program. The voxel size of egsphant file was 2.5 × 2.5 × 2.5 mm^3^. Air, lung, tissue, and bone were used as materials for converting images of the planning CT according to the previous study.^26^ The conversion of CT values to materials and electron density was performed using the calibration curve incorporated in DOSXYZnrc.^28^ Furthermore, the structure of treatment couch top, which was incorporated in a treatment planning system (TPS) (Eclipse version 13.0, Varian Medical Systems, Inc., Palo Alto, CA) was inserted into the egsphant file. The material of the treatment couch top was selected based on the CT values obtained according to a previous study.^29^, ^30^ These values were assigned to the tissue and lung materials according to the calibration curve in DOSXYZnrc. For the transport parameter of EGSnrc, we set the same ECUT, PCUT and parameter setting as those used for the simulations described in section 2.A. The number of histories was 2 × 10^10^ to obtain a statistical uncertainty below 3%. The simulation was performed with source of 20 (phase‐space source through dynamic library with multiple variable geometry setting) and the calculation point was set from −180° to 180° with increments of 2°. This source could simulate continuous motion of the phase‐space source relative to the phantom over multiple incident directions. Each calculation took approximately 40 h on a single‐CPU workstation. The simulated dose was defined as.

(2) $$D_{\text{abs}}=D_{\text{MC}}\times f_{\text{cal}}\times N\times A\times T_{\text{acq}}\times F\times T_{\text{pulse}}$$

where $f_{\text{cal}}$ is the calibration factor calculated at a depth of 2 cm using equation (1), *N* is the number of CBCT acquisitions, *A* is the tube current, $T_{\text{acq}}$ is the acquisition time, *F* is the frame rate during CBCT acquisition, and $T_{\text{pulse}}$ is the X‐ray pulse duration per frame. In this study, we calculated the absolute dose for 3D‐CBCT (thorax and pelvis modes) and 4D‐CBCT acquisition mode to evaluate the impact of the acquisition mode. The total acquisition mAs value was 360, 1440, and 672 mAs for thorax, pelvis, and 4D‐CBCT acquisition mode. For the 4D‐CBCT, the same acquisition protocol was used for thorax and liver. We set *A* to 20, 80, and 40 mA, *T* to 60, 60, and 120 s, *F* to 15, 15, and 7 frames/s for thorax, pelvis, and 4D‐CBCT acquisition modes, respectively, *N* to 1, and $T_{\text{pulse}}$ to 20 ms, for all acquisition modes. We used single $f_{\text{cal}}$ value in all the simulations, and $f_{\text{cal}}$ set it to 5.91 × 10^15^ Gy^2^/mAs based on eq. (1).

Statistical analyses were performed with a one‐way analysis of variance followed by the Tukey‐Kramer post hoc test. P‐values of less than 0.05 were considered statistically significant.

The absolute dose files were converted into DICOM‐RT dose file formats that contained the imaging dose data by using an in‐house program.^31^ For the conversion, the absolute dose data were used as pixel data of the DICOM‐RT dose file. To obtain the dose data with mGy, the absolute dose data were adjusted when converting into DICOM‐RT. The structures for lung, stomach, bone marrow, esophagus, liver, thyroid, bone surface, skin, adrenal glands, gallbladder, heart, intestine, kidney, pancreas, and spleen were contoured using TPS. The converted DICOM‐RT dose files were imported to the TPS, which was used to perform the data analysis. The mean doses to each organ were calculated in the TPS using imported DICOM‐RT dose file and contoured structures.

### 2.4 Equivalent and effective dose calculations

For each patient, both the organ equivalent dose and effective dose for 4D‐CBCT acquisitions were calculated to evaluate the imaging dose and its biological effect. The equivalent doses to the contoured organs were calculated using the corresponding mean doses and radiation weighting factors. The effective dose, *E*, for the 4D‐CBCT acquisition was defined as.

(3) $$E=\sum_{T}w_{T}\sum_{R}w_{R}D_{T,R}$$

where $w_{T}$ is the weighting factor of tissue *T*, $w_{R}$ is the radiation weighting factor, and $D_{T,R}$ is the mean absorbed dose to tissue *T*. The weighting factors used are based on publication 103 of the International Commission on Radiological Protection (ICRP),^32^ and the radiation weighting factor of a photon was considered as 1.0 in this study. In addition, the patient’s body mass index (BMI) was divided into three classes (underweight: < 18.50 kg/m^2^, normal‐weight: 18.50–24.99 kg/m^2^, overweight: ≥ 25.00 kg/m^2^) according to the BMI classification of World Health Organization.^33^ The effective dose for each BMI class was calculated for the lung and liver cancer patients to evaluate the influence of body size.

### 2.5 EAR calculation

The EAR was calculated to determine the secondary cancer incidence for thorax, pelvis and 4D‐CBCT acquisition modes based on the simulated mean dose to each organ. The EAR to skin, lung, thyroid, liver, kidney, esophagus, stomach, pancreas, and intestines was calculated based on BEIR VII model.^34^ The BEIR VII model is defined as.

(4) $${\text{EAR}}_{\text{BEIR}}=\beta_{M/F}\cdot D_{T,R}\cdot \exp\left(\gamma \cdot \frac{e-30}{10}\right)\cdot {\left(\frac{A}{A_{0}}\right)}^{\eta }$$

where $D_{T,R}$ is the mean absorbed dose to tissue *T*, *e* is the age at radiation exposure, *A* is the attained age of the individual or population under consideration and *A_0_* is the age with which the EAR models are standardized. Parameters $\beta_{M/F}$, γ, and η, are organ specific values in the BEIR VII report. Additionally, $\beta_{M}$ is the parameter for males, and $\beta_{F}$ is the parameter for females, while *A_o_* was set to 60 based on the BEIR VII report. In this study, we set *A* to 70 yr, and *e* to 30, 40, 50, and 60 yr, to evaluate the impact of time since radiation exposure.

## 3 RESULTS

### 3.1 Validation of Monte Carlo simulation for OBI

**Figure 2:**
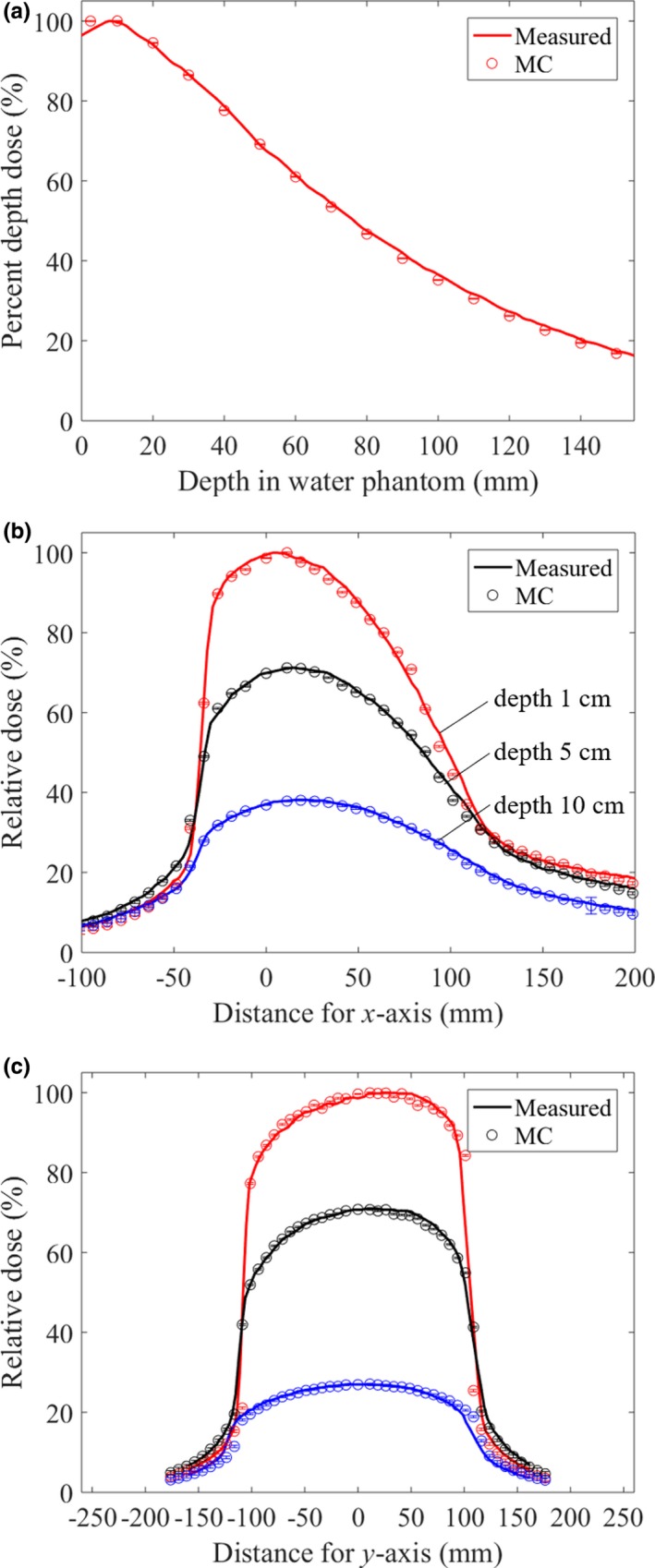
Comparison between simulated and measured percent depth dose (PDD) at the isocenter, and OCRs at the depths of 1, 5, and 10 cm, along the *x*‐ and *y*‐axes. (a) Simulated and measured PDDs agree within 2% at every depth. (b) Simulated and measured OCRs along the *x*‐axis agree within 2%, except around the penumbra. (c) The simulated and measured OCRs along the *y*‐axis also agree within 2%, except around the penumbra.

Figure 2(a) shows that the simulated and measured PDD in the 3D water phantom agree within 2% at every depth. Figures 2(b) and 2(c) show that the simulated and measured OCRs at depths of 1, 5, and 10 cm along the *x*‐ and *y*‐axes also agree within 2% at each depth, except around the penumbra, and exhibit asymmetric profiles along both axes.

### 3.2 Estimation of imaging dose in 4D‐CBCT acquisition for clinical cases

**Figure 3:**
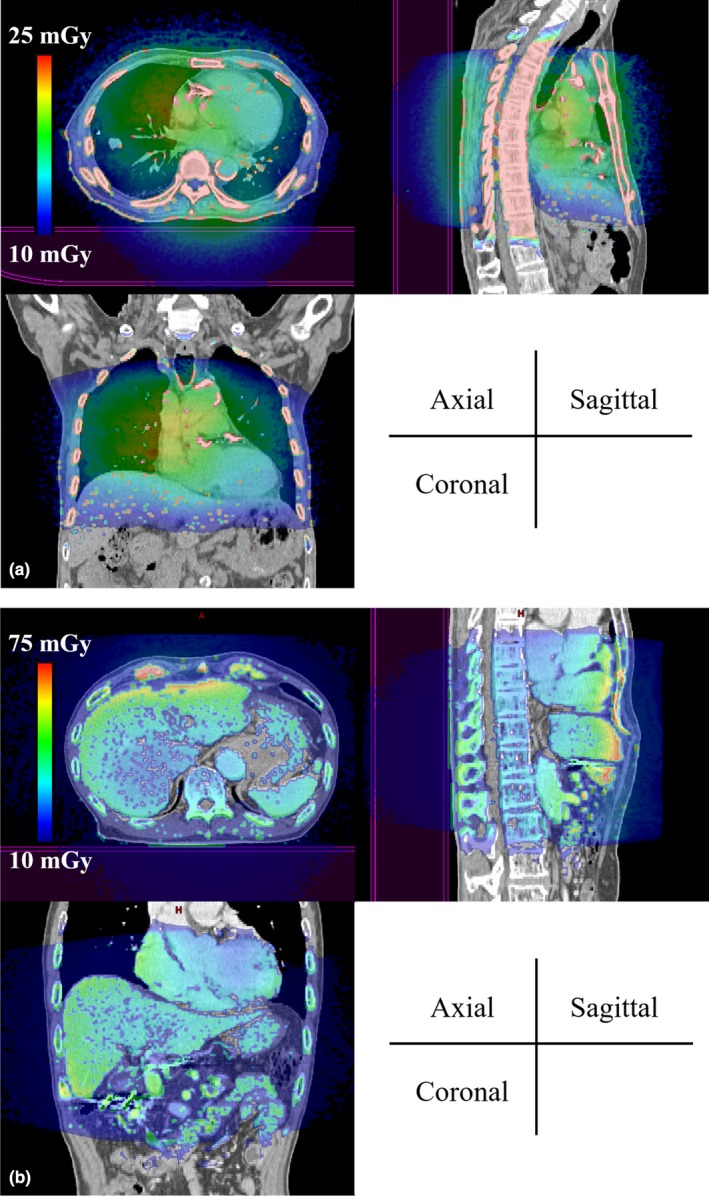
Estimated imaging dose distribution for lung and liver cancer patients. (a) For lung cancer patient 4, the high‐dose region is distributed from the skin to the mediastinum. (b) For liver cancer patient 9, the high‐dose region is distributed around the liver surface.

Figures 3(a) and 3(b) show the dose distributions obtained from 4D‐CBCT acquisitions in color wash at the isocenter for lung cancer patient 4 and liver cancer patient 9. For the lung cancer patient, the high‐dose region is distributed from the skin to the mediastinum, whereas for the liver cancer patient, the high‐dose region is distributed around the liver surface.

Table 3 shows the mean equivalent doses and mean effective doses of the 15 patients studied herein for each acquisition mode. For the lung cancer patients, the equivalent doses to the lung are 7.6 ± 1.2, 30.5 ± 4.6, and 14.3 ± 2.2 mSv, and for the liver cancer patients, the equivalent doses to the liver are 12.8 ± 3.0, 51.3 ± 12.1, and 24.0 ± 5.6 mSv for the respective thorax, pelvis, and 4D‐CBCT acquisition mode. For 4D‐CBCT of the lung cancer patients, the equivalent doses to esophagus, heart, and thyroid are higher than 10 mSv except for the dose to the bone, whereas for the 4D‐CBCT of the liver cancer patients, the equivalent doses to the heart, spleen, and pancreas, are higher than 20 mSv. The mean effective doses are 3.9 ± 0.5, 15.7 ± 2.0, and 7.3 ± 0.9 mSv, in the case of the lung cancer patients, and 4.2 ± 0.6, 16.7 ± 2.4, and 7.8 ± 1.1 mSv, in the case of the liver cancer patients for the thorax, pelvis and 4D‐CBCT acquisition mode, respectively. There are significant differences between the mean effective doses of 3D‐CBCT and 4D‐CBCT. The effective dose of 4D‐CBCT is significantly higher than that of the thorax mode (*P* < 0.05), and is significantly lower than that of the pelvis mode (*P* < 0.05). In the case of the lung cancer patients, the differences between the thorax and 4D‐CBCT modes, and pelvis and 4D‐CBCT modes are 3.4 and 8.4 mSv, respectively. In the case of liver cancer patients, the differences between the thorax and 4D‐CBCT modes, and the pelvis and 4D‐CBCT modes are 3.6 and 8.9 mSv, respectively. The effective doses of 4D‐CBCT for lung cancer patients are almost the same as that for liver cancer patients.

**Table 3:** Organ equivalent dose (mSv) and effective dose for 3D‐CBCT (thorax, pelvis) and 4D‐CBCT modes.

Organ (weighting factor)	Organ volume (cm^3^)	Lung cancer patients			Liver cancer patients		
		3D‐CBCT (Thorax mode)	3D‐CBCT (Pelvis mode)	4D‐CBCT	3D‐CBCT (Thorax mode)	3D‐CBCT (Pelvis mode)	4D‐CBCT
Lung (0.12)	2259.0 ± 722.5	7.6 ± 1.2	30.5 ± 4.6	14.3 ± 2.2	4.2 ± 0.9	16.7 ± 3.6	7.8 ± 1.7
Stomach (0.12)	229.4 ± 175.7	3.6 ± 2.1	14.3 ± 8.2	6.7 ± 3.8	9.6 ± 2.6	38.6 ± 10.3	18.0 ± 4.8
Bone marrow (0.12)	324.6 ± 118.1	10.8 ± 2.2	43.1 ± 8.7	20.2 ± 4.1	8.1 ± 3.3	32.4 ± 13.4	15.1 ± 6.2
Esophagus (0.04)	33.7 ± 13.1	7.5 ± 1.8	29.9 ± 7.2	14.0 ± 3.3	4.7 ± 0.9	18.9 ± 3.8	8.8 ± 1.8
Liver (0.04)	1049.4 ± 218.9	4.9 ± 2.3	19.6 ± 9.3	9.1 ± 4.4	12.8 ± 3.0	51.3 ± 12.1	24.0 ± 5.6
Thyroid (0.04)	18.4 ± 15.5	10.1 ± 10.8	40.2 ± 43.2	18.8 ± 20.2	–	–	–
Bone surface (0.01)	990.5 ± 321.0	12.7 ± 2.2	51.0 ± 8.6	23.8 ± 4.0	10.7 ± 2.5	42.8 ± 10.0	20.0 ± 4.7
Skin (0.01)	931.4 ± 179.5	4.4 ± 0.6	17.6 ± 2.6	8.2 ± 1.2	5.2 ± 1.2	20.9 ± 4.8	9.8 ± 2.2
Adrenal glands^a^	3.0 ± 1.6	2.8 ± 2.1	11.2 ± 8.5	5.2 ± 4.0	8.7 ± 2.3	34.8 ± 9.0	16.3 ± 4.2
Gallbladder^a^	12.7 ± 13.1	2.2 ± 2.0	8.8 ± 7.9	4.1 ± 3.7	8.6 ± 3.9	34.3 ± 15.6	16.0 ± 7.3
Heart^a^	712.3 ± 263.5	8.5 ± 2.1	34.2 ± 8.2	16.0 ± 3.8	13.1 ± 3.1	52.3 ± 12.3	24.5 ± 5.8
Intestine^a^	556.0 ± 262.7	1.5 ± 0.8	5.9 ± 3.2	2.7 ± 1.5	5.8 ± 2.1	23.2 ± 8.3	10.8 ± 3.9
Kidney^a^	251.9 ± 79.6	1.3 ± 1.1	5.4 ± 4.5	2.5 ± 2.1	10.1 ± 3.4	40.5 ± 13.7	18.9 ± 6.4
Pancreas a	32.1 ± 14.1	2.3 ± 1.8	9.1 ± 7.2	4.2 ± 3.4	13.5 ± 3.5	54.1 ± 14.0	25.3 ± 6.5
Spleen^a^	129.5 ± 85.8	4.4 ± 3.0	17.6 ± 11.9	8.2 ± 5.6	14.9 ± 2.9	59.5 ± 11.6	27.8 ± 5.4
Effective dose (mSv)		3.9 ± 0.5	15.7 ± 2.0	7.3 ± 0.9	4.2 ± 0.6	16.7 ± 2.4	7.8 ± 1.1

4D‐CBCT, four‐dimensional cone‐beam computed tomography.

a The weighting factor of remainder tissue, 0.12, applied to the arithmetic mean dose of the 13 organs and tissues decided in ICRP 103. To calculate the effective dose, we calculated the arithmetic mean dose of remainder tissue, and multiplied by the tissue weighting factor.

**Figure 4:**
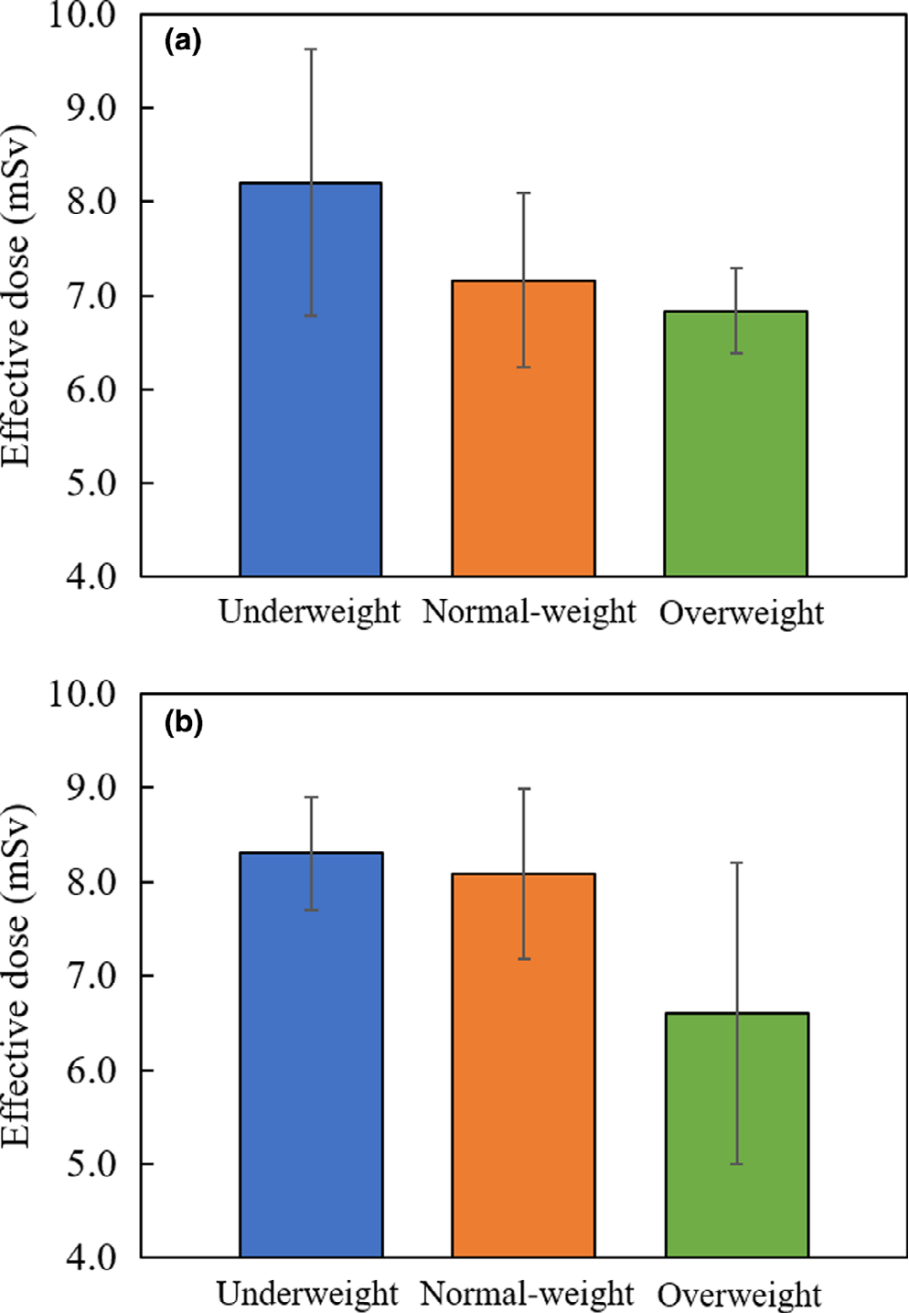
Mean effective dose of underweight (<18.50 kg/m^2^), normal‐weight (18.50–24.99 kg/m^2^), and overweight (≥25.00 kg/m^2^) patients for (a) lung and (b) liver cancer patients. For lung patients, the mean effective doses were 8.2 ± 1.4, 7.2 ± 0.9, and 6.8 ± 0.5 for underweight, normal‐weight, and overweight, respectively. For liver patients, the mean effective doses were 8.3 ± 0.6, 8.1 ± 0.9, and 6.6 ± 1.6 for underweight, normal‐weight, and overweight, respectively.

Figure 4 shows the effective dose of 4D‐CBCT acquisition for each BMI class. The effective dose showed a tendency to decrease as the BMI increased. For the BMI classification of the lung cancer patients, three patients were underweight, eight patients were normal‐weight, and the rest were overweight. The mean effective doses were 8.2 ± 1.4, 7.2 ± 0.9, and 6.8 ± 0.5 mSv for underweight, normal‐weight, and overweight class, respectively. For the BMI classification of liver cancer patients, two patients were underweight, ten patients were normal‐weight, and the rest were overweight. The mean effective doses were 8.3 ± 0.6, 8.1 ± 0.9, and 6.6 ± 1.6 mSv for underweight, normal‐weight, and overweight class, respectively.

Table 4 lists the EAR of secondary cancer incidence for the thorax, pelvis, and 4D‐CBCT modes. For the EAR of each acquisition mode, the exposure age was considered to be 30 yr and the age for cancer incidence was considered to be 70 yr. For the lung cancer patients, the lung EAR is 7.3 and 10.8 cases per million person‐years, while for the thyroid, esophagus, and stomach the EARs are 12.9 and 10.6, 9.6 and 7.9, 6.3 and 6.2 cases per million person‐years for males and females, respectively. For the liver cancer patients, the liver EAR is 9.9 and 4.5 cases per million person‐years, and for the stomach, kidney, and pancreas the EARs are 16.7 and 16.9, 12.9 and 10.7, 17.3 and 14.2 cases per million person‐years for males and females, respectively. Furthermore, EAR increases with increasing doses to the organ, but the EARs for pelvis acquisition modes are larger than the thorax and 4D‐CBCT acquisition modes.

**Table 4:** EAR of secondary cancer incidence for 3D‐CBCT (thorax, pelvis) and 4D‐CBCT modes.

Organ	*β*_M_/*β*_F_	*γ*	*η*	EAR of secondary cancer incidence (cases per million person‐years)					
				Lung cancer patients			Liver cancer patients		
				3D‐CBCT (Thorax mode)	3D‐CBCT (Pelvis mode)	4D‐CBCT	3D‐CBCT (Thorax mode)	3D‐CBCT (Pelvis mode)	4D‐CBCT
Lung	2.3/3.4	–0.41	5.2	3.9/5.8	15.7/23.1	7.3/10.8	2.1/3.2	8.5/12.6	4.0/ 5.9
Stomach	7.0/7.1	0.002	1.8	3.3/3.3	13.2/13.4	6.2/6.2	8.9/9.0	35.7/36.2	16.7/16.9
Esophagus^a^	5.1/4.2	–0.39	1.9	5.1/4.2	20.4/16.8	9.6/7.9	3.2/2.7	12.9/10.7	6.0/5.0
Liver	2.2/1.0	–0.41	4.1	2.0/0.9	8.1/3.7	3.8/1.7	5.3/2.4	21.2/9.6	9.9/4.5
Thyroid^a^	5.1/4.2	–0.39	1.9	6.9/5.7	27.5/22.7	12.9/10.6	–	–	–
Skin^a^	5.1/4.2	–0.39	1.9	3.0/2.5	12.0/9.9	5.6/4.6	3.6/2.9	14.3/11.8	6.7/5.5
Intestine	2.2/0.84	–1.00	5.7	0.8/0.3	3.1/1.2	1.4/0.6	3.1/1.2	12.3/4.7	5.7/2.2
Kidney^a^	5.1/4.2	–0.39	1.9	0.9/0.8	3.7/3.0	1.7/1.4	6.9/5.7	27.7/22.8	12.9/10.7
Pancreas^a^	5.1/4.2	–0.39	1.9	1.5/1.3	6.2/5.1	2.9/2.4	9.2/7.6	37.0/30.4	17.3/14.2

a The values of parameter of $\beta_{M/F}$, γ, and η were based on other solid cancer values listed in BEIR VII report.

**Figure 5:**
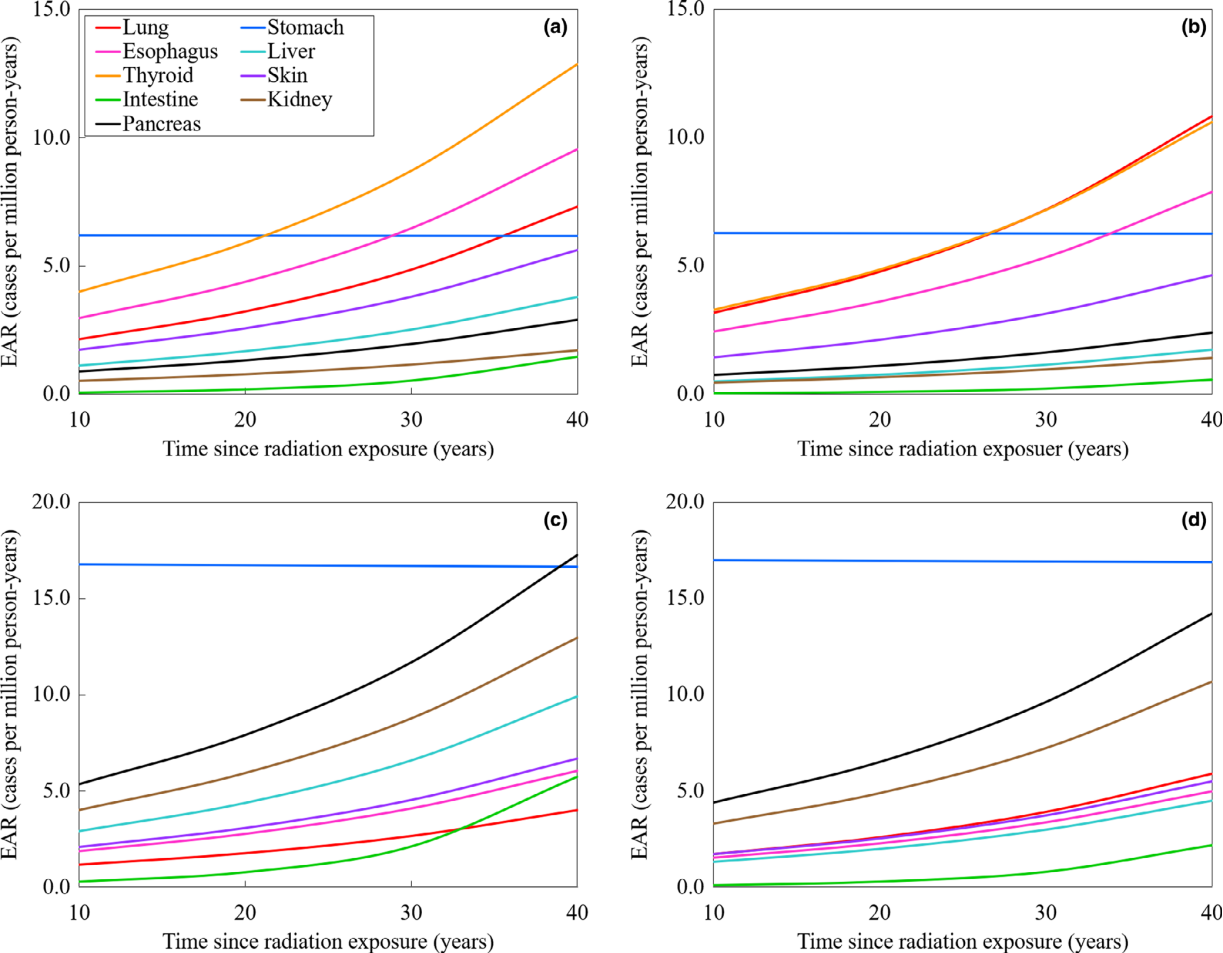
Variation of EAR as a function of time since radiation exposure showing increasing trends. The EAR shows different trends between males and females. For (a) male and (b) female lung cancer patients, thyroid shows the highest EAR among the organs evaluated in this study. For (c) male and (d) female liver cancer patients, the stomach EAR always shows highest EAR among organs/tissues. The stomach EAR is not shown to be affected by time in all cases. EAR, excess absolute risk.

Figure 5 shows the function of EAR for 4D‐CBCT over time since radiation exposure. As observed, the EARs increase as a function of time since radiation exposure. The EAR change shows different trends for males and females. In the case of lung cancer patients, thyroid shows the highest EAR among the organs evaluated in this study. In the case of males and females, thyroid EAR is 4.0, 5.9, 8.7, and 12.9 cases per million person‐years, and 3.3, 4.9, 7.2, and 10.6 cases per million person‐years at 10, 20, 30, and 40 yr since radiation exposure, respectively. In the case of the liver cancer patients, the stomach EAR is always the highest among all the organs. The stomach EAR does not change with time, and is approximately 17 cases per million person‐years, regardless of the time since radiation exposure.

## 4 DISCUSSION

In this study, we estimated the organ equivalent dose, effective imaging dose, and EAR of secondary cancer incidence during 4D‐CBCT acquisition for clinical cases using a Monte Carlo simulation.

The OBI was configured as shown in Fig. 2, and asymmetric OCRs were obtained along the *x*‐axis using the half‐bowtie filter and along the *y*‐axis by the heel effect. The heel effect occurred as a result of the geometry of the anode. The heel effect showed a lower x‐ray intensity toward the anode end and a higher x‐ray intensity toward the cathode end. For the OBI in this study, the anode‐cathode direction was positioned along the *y*‐axis, and thus the heel effect occurred along the *y*‐axis.

Table 5 shows the outcomes from imaging dose studies for IGRT. For the CBCT, many studies have evaluated the effective dose using various methods. Aduhaimed et al. simulated the effective dose of OBI using Monte Carlo simulation.^35^ Kan et al. and Dzierma et al. measured the effective dose of CBCT using a thermo luminescence dosimeter (TLD).^16^, ^19^ The effective doses of thorax mode in our results are comparable to those of 3D‐CBCT for lung in previous studies. The effective doses of the pelvis mode in our results are one and half to three times greater than those of 3D‐CBCT for the abdominal and pelvic region in previous studies. In addition, the effective doses of 4D‐CBCT are one and half to two times greater than those of 3D‐CBCT in previous studies. The effective dose depends on acquisition parameters such as mAs value, thus, the 4D‐CBCT tends to have greater effective dose than 3D‐CBCT. Vergalasova et al. reported that 3D‐CBCT underestimates ITV by 24.2–40.1%, depending on the tumor size and pattern of respiratory motion. In contrast, 4D‐CBCT can estimate ITV with high accuracy.^36^ Although the effective dose of 4D‐CBCT is greater than that of 3D‐CBCT, 4D‐CBCT can estimate the target position and volume with high accuracy. Therefore, it is suitable for thoracic and abdominal regions with respiratory motion.

**Table 5:** Summary of effective imaging doses from image‐guided radiotherapy (IGRT).

Study	Modality	Method	Protocol	Region	kV	mAs	Effective dose (mSv)
Aduhaimed et al. 35	Varian OBI	BEAMnrc/DOSXYZnrc	CBCT	Lung (male)	125	270	3.34
				Lung (female)	125	270	3.97
				Pelvis (male)	125	1080	6.05
				Pelvis(female)	125	1080	11.30
Dzierma et al. 19	Siemens Artiste	Measurement using TLD	CBCT (normal)	Abdomen	121	306.5	3.75
			CBCT (high quality)	Abdomen	121	799.2	9.16
Kan et al. 16	Varian OBI	Measurement using TLD	CBCT (low dose)	Lung	125	264	5.23
			CBCT (low dose)	Pelvis	125	264	4.89
Marchant and Joshi 37	Elekta XVI	Geant4 for tomographic emission	4D‐CBCT	Lung (female)	120	312	8.30
				Lung (male)	120	312	7.88
This study	Varian OBI	BEAMnrc/DOSXYZnrc	CBCT (thorax mode)	Lung	125	360	3.9 ± 0.5
				Liver	125	360	4.2 ± 0.6
			CBCT (pelvis mode)	Lung	125	1440	15.7 ± 2.0
				Liver	125	1440	16.7 ± 2.4
			4D‐CBCT	Lung	125	624	7.3 ± 0.9
				Liver	125	624	7.8 ± 1.1

CBCT, cone‐beam computed tomography; OBI, on‐board imager; TLD, thermo‐luminescent dosimeter; XVI, X‐ray volume imaging.

Marchant and Joshi estimated the effective imaging dose of CBCT acquisition using an X‐ray volume imaging (XVI) system (Elekta AB, Crawley, UK). The effective doses of 4D‐CBCT lung protocol in the XVI system were 8.30 and 7.88 mSv for the male and female lung phantoms.^37^ Our result showed that the effective dose for 4D‐CBCT was comparable to the dose for 4D‐CBCT in those reports. However, these authors evaluated the imaging dose in the phantoms; they could not evaluate clinical patients. In addition, only a few prior studies have investigated the imaging dose of 4D‐CBCT in clinical cases. Our results will contribute to the clarification of the imaging dose of 4D‐CBCT in clinical cases.

For the comparison between 3D‐CBCT and 4D‐CBCT, the effective dose of pelvis mode was approximately two times higher than that of 4D‐CBCT, and the effective dose of 4D‐CBCT was significantly higher than that of thorax mode. The effective dose for CBCT varied depending on the acquisition parameters, and thus it is important that the acquisition mode and acquisition parameter are appropriately selected. The CT dose index (CTDI) is often used to evaluate the imaging dose for CT and CBCT. Although CTDI can evaluate the imaging dose easily, it cannot evaluate the dose to the organ for each acquisition technique. Our results contributed to the determination of more realistic imaging doses than CTDI in clinical cases. In this sense, more detailed information for the imaging dose to organs may be obtained using our results.

A high correlation between the effective imaging dose of the CBCT acquisition and the size of the chest circumference has been reported by Zhang et al.,^38^ where the dose decreased with increasing chest circumferences. These results suggest that the effective dose of 4D‐CBCT acquisition should also be dependent on the patient’s chest circumference. Similarly, Hwang et al. reported a high correlation between the effective imaging dose of angiographic CBCT acquisition and patient BMI.^39^ In our study, the effective dose of 4D‐CBCT acquisitions for each BMI class followed the same trend (Fig. 4), suggesting that the imaging dose decreases as the BMI increases. Additionally, it is clear that the effective dose increases with the organ dose increasing (Table 3), and thus the EAR for each BMI class followed the same trend as effective for BMI class. If the acquisition protocol for normal‐weight patients is used for all patients, underweight patients will receive excessive imaging dose. In contrast, the image quality of 4D‐CBCT of overweight patients might degrade due to insufficient imaging dose (Fig. 4). To avoid excessive/insufficient imaging dose, acquisition parameters such as mAs value could be optimized using our results. For underweight lung cancer patients, mAs values can be reduced by 14%, compared with the acquisition protocol for normal‐weight patients to avoid excessive doses. For overweight lung cancer patients, mAs values can be increased by 3%, compared with the acquisition protocol for normal‐weight patients to avoid image quality degradation. Similarly, for underweight liver cancer patients, mAs values can be reduced by 4%. For overweight liver cancer patients, mAs values can be increased by 19%. In this study, the effective dose of 4D‐CBCT acquisition for each BMI class was evaluated. The number of patients for each BMI class was small, thus, there might be some statistical uncertainty. However, for clinical cases, our results will contribute to the optimization of the acquisition parameter of 4D‐CBCT while considering BMI.

Regarding the risk of secondary cancer incidences, Dzierma et al. calculated the EAR of several CT and CBCT exams for abdominal organs.^19^ The liver and stomach EARs were, respectively, 0.13 and 0.39 cases per million person‐years using the normal full‐scan mode, and 0.34 and 1.02 cases per million person‐years using the high‐quality full‐scan mode. Our results showed that the EARs were approximately 10 times higher compared to their report. As can be observed in Fig. 5, almost all of the EARs increased as a function of time since radiation exposure. However, the stomach EAR was constant regardless of time. On the other hand, the intestine EAR increased suddenly at 30 yr after radiation exposure, especially in male liver cancer patients. The function of EAR over time depends on parameter *γ* according to Eq. (4). In the cases of the stomach and intestine, the parameter of *γ* was different from other organs. Accordingly, different EAR results were obtained compared to other organs. Therefore, it is possible that EARs are unexpectedly high depending on the organ dose and radiation exposure age. Thus, we should set the appropriate scan range to reduce the unnecessary cancer incidence risk. Furthermore, we should use 4D‐CBCT based on the consideration of the age of the patients in clinical cases. Kim et al. have reported that secondary cancer risk after radiotherapy.^40^ Although the EAR for 4D‐CBCT is smaller than their results, we should manage the imaging dose to normal tissue to reduce the risk of secondary cancers.

During radiotherapy of the lung and upper abdominal region, 4D‐CBCT suitably manages respiratory motion as its resulting images can reduce both the internal margins of the target and the toxicity to normal tissue. The ICRP publication 26 recommends that the patient radiation dose should be minimized to a level as low as can be reasonably achievable (ALARA).^41^ This concept is also applicable to 4D‐CBCT, as it effectively manages respiratory motion during stereotactic body radiotherapy by delivering a high‐dose to the target with few fractions. Furthermore, ICRP publication 118 recommends that the radiation exposure dose should be managed to be less than 0.5 Gy to avoid the normal tissue reaction such as cataract, circulatory disease and bone marrow suppression.^42^ In this study, the maximum equivalent doses to the bone marrow and the heart were respectively 20.2 and 24.5 mSv. According to the recommendation, the number of 4D‐CBCT acquisitions should be <20 times. Therefore, 4D‐CBCT is not suitable for conventional external beam radiotherapy, and imaging doses that exceed the recommendation, especially for multiple acquisitions, should be avoided in clinical cases. The imaging dose and EAR of secondary cancer incidence increase proportionally with the number of 4D‐CBCT acquisitions. When we acquired the 4D‐CBCT for every fraction in conventional external beam radiotherapy, the imaging dose and EAR were higher than our results.

To reduce both the effective dose and EAR, the imaging dose per acquisition should be reduced. Equation (2) shows that the absolute dose is proportional to the tube current and acquisition times. In the case of 4D‐CBCT, we can select the rotational speed of the gantry from 1 to 6°/s to reduce the tube current and acquisition time. If we set the gantry rotation speed to 6°/s, the acquisition time is set to 60 s, and the effective dose and EAR are almost the same as those used for thorax acquisition mode in our study. However, some studies have reported that there is a correlation between the gantry speed and image quality in 4D‐CBCT.^8^, ^9^ They showed that a high‐speed gantry rotation scan degrades the image quality and accuracy to track tumor motion trajectories, and pointed out that a high‐speed gantry rotation can be avoided during 4D‐CBCT acquisition. Therefore, we should optimize only the tube current. However, the tube current reduction induces image quality degradation. Hao et al. suggested that the image quality of low‐dose CBCT can be improved using iterative reconstruction.^43^ If we apply the technique to 4D‐CBCT, we could prevent image quality degradation and reduce the effective dose and EAR.

In this study, there were several organs/tissues for which the mean dose could not be evaluated, e.g. muscle, breast, lymph node, and thymus. We used planning CT images to simulate the organ dose, however, the imaging range did not contain all the organs that should be evaluated. Additionally, we could not delineate organs/tissues such as the lymph nodes and the thymus, because some organs/tissues have low contrast in CT images. Therefore, the effective dose may be underestimated. For the calculation of the effective dose, the mean absorbed dose for the 12 organs could not be acquired in this study. However, the doses to the gonads, bladder, brain, salivary glands, uterus, oral mucosa and prostate are close to 0 mSv. Thus, the dose to organs described above can be ignored. For the remaining five organs/tissues such as the breast, muscle, thymus, lymph nodes and extra thoracic region, we calculated the effective dose to assign the mean absorbed dose of these organs/tissues to those of nearby organs, whereby the effective dose was 20% larger than our results. Thus, the underestimation of the effective dose in this study was approximately 20%. Furthermore, the whole intestine for lung cancer patients, and whole lung and intestine for liver cancer patients were not included in the planning CT imaging range. The mean dose to these organs might be overestimated, and thus the effective dose and EAR might be overestimated. For the calculation of the effective dose, the weighting factors of the lung, liver and intestine were 0.12, 0.04, and 0.12. Thus, it is possible that we overestimated the effective dose by approximately 20% for each patient.

To reduce the imaging dose and risk of secondary cancer incidence, we recommend optimization of the 4D‐CBCT parameters and not applying excessive acquisitions for patients.

## 5 CONCLUSION

In this study, we evaluated the organ equivalent dose, effective imaging dose, and risk of secondary cancer incidence for 4D‐CBCT acquisition in clinical cases. The effective dose for 4D‐CBCT was two times larger than that for thorax acquisition modes. Furthermore, the risk of secondary cancer incidence varied depending on the acquisition parameter, the time since radiation exposure and the number of 4D‐CBCT acquisitions. For clinical cases, we should use 4D‐CBCT with consideration for the effective dose and risk of secondary cancer incidence. Our results contributed to the determination of the acquisition parameters and frequency of 4D‐CBCT acquisitions in clinical cases.

## CONFLICT OF INTEREST

The authors have no conflict of interest.
